# Catalytic conversion of glucose to 5-hydroxymethylfurfural using zirconium-containing metal–organic frameworks using microwave heating†

**DOI:** 10.1039/c8ra06021e

**Published:** 2018-09-10

**Authors:** Jue Gong, Michael J. Katz, Francesca M. Kerton

**Affiliations:** Department of Chemistry, Memorial University of Newfoundland 230 Elizabeth Avenue St. John's NL A1B 3X7 Canada fkerton@mun.ca mkatz@mun.ca

## Abstract

5-Hydroxymethylfurfural (5-HMF) can be prepared by the catalytic dehydration of glucose or fructose using a range of homogeneous or heterogeneous catalysts. For our research, a selection of closely related Metal–Organic Frameworks (MOFs) were used as catalysts in the conversion of glucose to 5-HMF due to their chemical and thermal stability as well as the Lewis acidity of zirconium. Our initial study focused on the use of UiO-66–X (X = H, NH_2_ and SO_3_H), optimization of the dehydration reaction conditions, and correlation of the catalytic activity with the MOF's properties, in particular, their surface area. The highest yield of 5-HMF (28%) could be obtained using UiO-66 under optimal reaction conditions in dimethylsulfoxide and this could be increased to 37% in the presence of water. In catalyst recycling tests, we found the efficiency of UiO-66 was maintained across five runs (23%, 19%, 21%, 20%, 22.5%). The post-catalysis MOF, UiO-66–humin, was characterized using a range of techniques including PXRD, FT-IR, ^13^C Solid State NMR and N_2_ gas adsorption. We continued to optimize the reaction using MOF 808 as the catalyst. Notably, MOF 808 afforded higher yields of 5-HMF under the same conditions compared with the three UiO-66–X compounds. We propose that this might be attributed to the larger pores of MOF 808 or the more accessible zirconium centres.

## Abstract 2

Glucose can be converted to the platform chemical 5-HMF in up to 37% yield using porous, recyclable catalysts.

## Graphical abstract

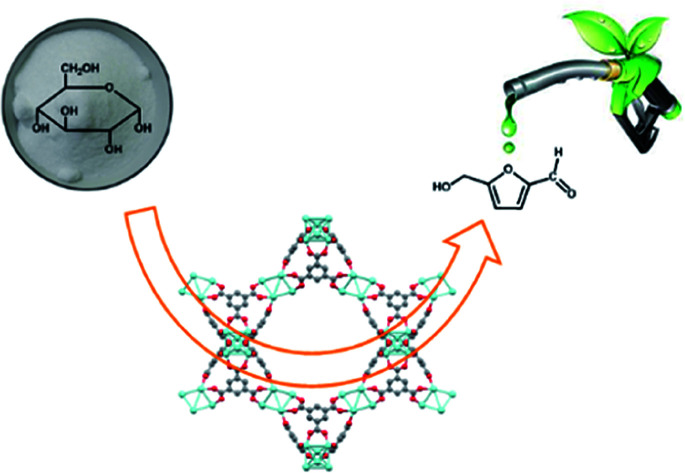

## Introduction

Production of CO_2_ from the combustion of fossil fuels and the resulting rise in atmospheric levels of CO_2_ is contributing to climate change.^1^ Thus, biomass is being explored as a renewable alternative to fossilized resources in a range of applications.^1,2^ Biomass from land plants is typically lignocellulosic biomass, which consists of cellulose, hemicellulose and lignin.^3^ Cellulose is a carbohydrate polymer containing hundreds to thousands of glucose units.^4^ Conversion of cellulose to valuable products, such as the platform chemicals 5-hydroxymethylfurfural and levulinic acid, is of particular interest.

**Fig. 1:**
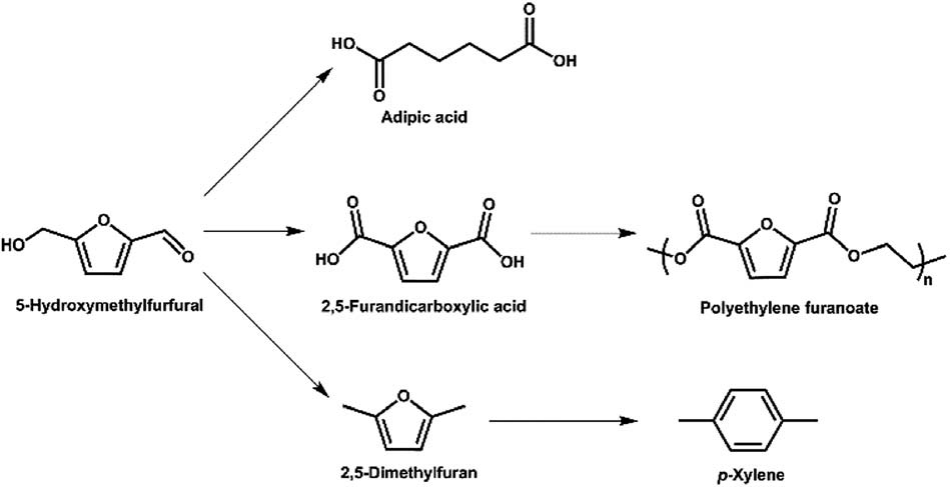
A range of applications for 5-HMF as a platform chemical.

5-Hydroxymethylfurfural (5-HMF) is an important platform chemical derived from glucose,^5,6^ and can be obtained in near quantitative yields using superior catalytic systems. It is a heterocyclic organic compound, containing aldehyde and alcohol functional groups in the 2,5 positions of a furan ring (Fig. 1). As further illustrated in Fig. 1, 5-HMF can be used as a building block for other compounds such as adipic acid (the precursor of nylon), 2,5-furandicarboxylic acid and *p*-xylene (*via* 2,5-dimethylfuran).^5,6^ The latter two compounds can be further converted (Fig. 1) to a broad range of valuable products including fuel additives and the bio-derived polymer polyethylene furanoate (PEF) that is a possible alternative to the widely-used petroleum-derived polymer polyethylene terephthalate (PET).^7^

Generally, 5-HMF can be synthesized *via* dehydration reactions of sugars such as glucose, fructose and sucrose with different homogeneous or heterogeneous catalysts.^5^ Several homogeneous catalysts (*e.g.*, H_2_SO_4_, HCl and CrCl_3_) have been investigated. However, difficulties in post-reaction catalyst separation prevents the further development of some of these systems.^8^ Thus, different heterogeneous catalysts such as metal–organic frameworks, metal oxides *e.g.* zirconias, zeolites and related porous materials *e.g.* mesoporous zirconium phosphates have begun to be investigated in recent years.^5,6^ In this way, it has become clear that a balance is needed between Lewis acidic sites to isomerize glucose to fructose and Brønsted acidic sites to dehydrate fructose to 5-HMF. Furthermore, the Lewis acidic sites can also lead to the formation of humin as a by-product.^6*a*,*d*^

Metal–organic frameworks (MOFs) are a class of crystalline materials, built up from metal cations/clusters (nodes) and bridging organic ligands (linkers).^9^ With judicious choice of node and linker, MOFs having a wide range of pore-sizes and pore functionalities have been formed.^10^ Additionally, pre/post synthetic functionalization of MOFs can be used to introduce different linkers or functionalities into the frameworks.^9*b*,11^ Given the toolbox available for MOF synthesis, different structures and properties can be designed. The combination of the facile synthesis as well as the large pore sizes, low density, and their thermal and chemical stability have made these materials ideal in many different fields such as gas storage and separation,^12^ catalytic reactions,^9*b*,13^ proton-, and ion-conduction.^14^

Currently, glucose conversion to 5-HMF with MOFs is in its infancy. Li, Hensen, and co-workers reported the first application of MOF (MIL-101) in dehydration of fructose and glucose. However, the yield was just 2% in dehydration of glucose to 5-HMF.^15^ Kitagawa's research group investigated isomerization from glucose to fructose using different MIL-101(Cr) derivatives and demonstrated that MIL-101(Cr)–SO_3_H had higher conversion and better selectivity.^16^ Bao and co-workers reported a 5-HMF yield of 44.9% for the dehydration of glucose to 5-HMF with MIL-101(Cr)–SO_3_H.^17^ Whilst Herbst and Janiak reported a 29% yield of 5-HMF with MIL-101(Cr)–SO_3_H in a solvent mixture, THF/H_2_O (v/v 39 : 1).^18^ Most recently, Katz, Farha and co-workers showed phosphate-modification in NU-1000 enabled the catalytic reaction of glucose to 5-HMF.^19^ In related research, the Zhao research group developed a new MOF, NUS-6, built from zirconium (Zr) or hafnium (Hf) clusters and sulfonated organic linkers.^20^ They examined these catalysts for dehydration of fructose to 5-HMF and found high yields (98%) and selectivity (98%) using NUS-6(Hf).^20^ However, it should be noted that they performed these reactions in DMSO and it is known that this solvent can catalyze fructose conversion to 5-HMF.^21^

Herein, a series of Zr-cluster-based MOFs were prepared, characterized and studied as catalysts for the conversion of glucose to 5-HMF. Initially, we performed catalytic reactions using UiO-66 and its analogues (UiO-66–NH_2_ and UiO-66–SO_3_H). We determined the optimal reaction conditions by varying different parameters such as time, temperature and catalyst loading. During this work, we found that the activity of UiO-66 was possibly inhibited due to the formation of humin. Humin, which is formed from acid-catalyzed dehydration of sugars, is a furanic, branched polymer and is often poorly characterized due to its intractable nature and limited solubility.^22^ Thus, a focus of our work was to characterize the humin formed using different methods (PXRD, FT-IR and ^13^C Solid State NMR). Furthermore, with optimized reaction conditions, we examined how larger pores within similar Zr-containing materials (MOF 808^23^) affect the catalytic conversion of glucose to 5-HMF.

## Experimental

### Instrumentation

FT-IR spectra (400–4000 cm^−1^) were recorded at room temperature on a Bruker Alpha FT-IR Spectrometer with a single-bounce diamond ATR accessory at a resolution of 4 cm^−1^ using 36 scans. Powder X-ray diffraction (PXRD) patterns were recorded with a Rigaku Ultima IV diffractometer equipped with a copper sealed-tube operated at 40 kV and 44 mA filtered to 1.54 Å using a graphite monochromator. Simulated powder diffractograms were obtained using the Mercury 3.8 software suite. N_2_ gas adsorption isotherms were collected on a Micrometrics Tristar II 3020 instrument with the sample maintained at 77 K using N_2(l)_. Before measurements, samples were activated on a Micrometrics Smart VacPrep by first heating at 353 K until a pressure <5 mmHg was achieved. Subsequently, the sample was heated under vacuum at 423 K for 10 h. Data was analyzed *via* the MicroActive Software suite. Thermogravimetric analysis (TGA) was carried out using a TA Instruments Q500. Samples were placed in a platinum pan and heated at a rate of 7 °C min^−1^ under a N_2_ atmosphere from 25–600 °C under a N_2_ atmosphere with flow rate of 50 mL min^−1^. Solid-state NMR spectra were obtained at 298 K using a Bruker Avance II 600 spectrometer, equipped with a SB Bruker 3.2 mm MAS triple-tuned probe operating at 600.33 MHz for ^1^H and 150.97 MHz for ^13^C. Chemical shifts were referenced to tetramethylsilane (TMS) using adamantane as an intermediate standard for ^13^C. The samples were spun at 20 kHz. ^13^C{^1^H} cross-polarization spectra were collected with a Hartmann–Hahn match at 62.5 kHz and ^1^H decoupling at 100 kHz. The recycle delay was 3 s and the contact time was 2000 ms. Solution ^1^H NMR experiments were performed on a Bruker AVANCE III 300.

### Synthesis of MOFs

UiO-66–X (X = H, NH_2_, SO_3_H) and MOF 808 were synthesized according to reported literature methods.^9*d*,23,24^ See the ESI† for details.

### Catalytic conversion of glucose to 5-HMF

Reactions were performed in triplicate, to assess reproducibility, using a Biotage microwave synthesizer. In a typical run, glucose (100 mg) and UiO-66 (20 mg) were weighed in a 2 mL microwave reaction vial. Subsequently, 2 mL DMSO-*d*_6_ was added. The vial was sealed and heated in the microwave at 160 °C for 30 min. After the reaction, the mixture was cooled with pressurized air to 50 °C. Then, the vial was removed from the synthesizer, allowed to cool to room temperature, opened, and 15 μL of 1-naphthaldehyde (internal standard) was added into the reaction mixture for quantitative ^1^H NMR analysis. Quantitative ^1^H NMR data was in agreement with data from GC analysis using a quantitation method previously reported.^24^

For the recycling test, a reaction was performed on a larger scale. Glucose (1000 mg), UiO-66 (200 mg), DMSO-*d*_6_ (15 mL) were added to a vial. The mixture was heated in the microwave at 160 °C for 30 min. After the reaction, the mixture was centrifuged and decanted out for quantitative ^1^H NMR analysis. The solid was washed with 15 mL DMSO (protio) three times and separated by vacuum filtration. Then, the solid was dried in a vacuum oven (10^−2^ mbar) at 80 °C overnight and reused for the next run. Alternatively, the solid was dried in a conventional oven at 200 °C overnight. Similar yields were obtained by each drying method.

For characterization of humin on the MOF surface, the reaction conditions and the treatment procedure of the used solid catalyst were the same as in the recycling test.

## Results and discussion

### Synthesis and characterization of MOFs

**Fig. 2:**
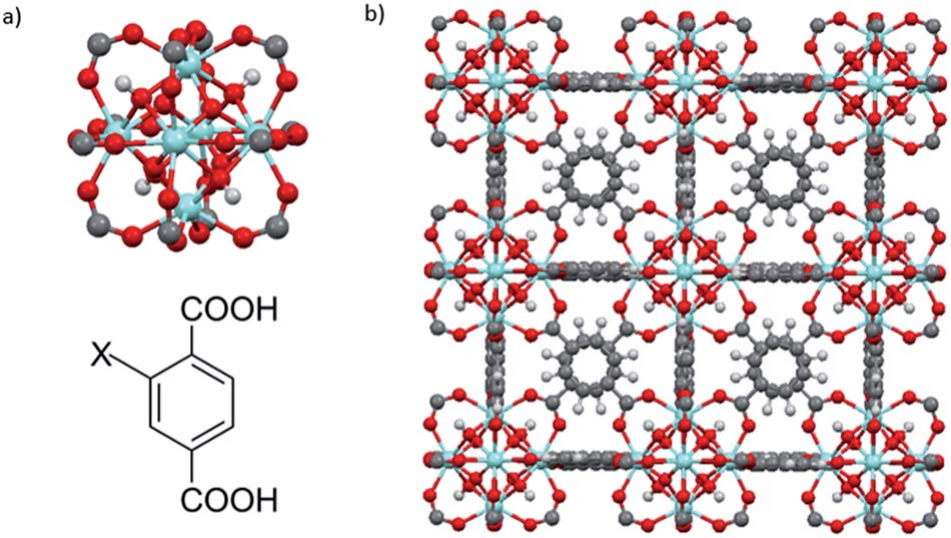
(a) The fundamental cornerstones of UiO-66 are Zr_6_O_4_(OH)_4_ octahedral clusters with twelve carboxylate groups coordinated to the zirconium cations (top) and 2-X-1,4-benzenedicarboxylate (BDC-X) organic ligands, X = H, NH_2_, or SO_3_H (bottom). (b) The cubic unit cell of UiO-66. Zr, blue; O, red; C, gray; H, white.

UiO-66–X (X = H, NH_2_, SO_3_H) were synthesized *via* a solvothermal method.^9*d*,25^ UiO-66–X consists of Zr_6_O_4_(OH)_4_ octahedral nodes with 2-X-1,4-benzenedicarboxylate (BDC-X) organic linkers (Fig. 2).^9*b*,25^

**Fig. 3:**
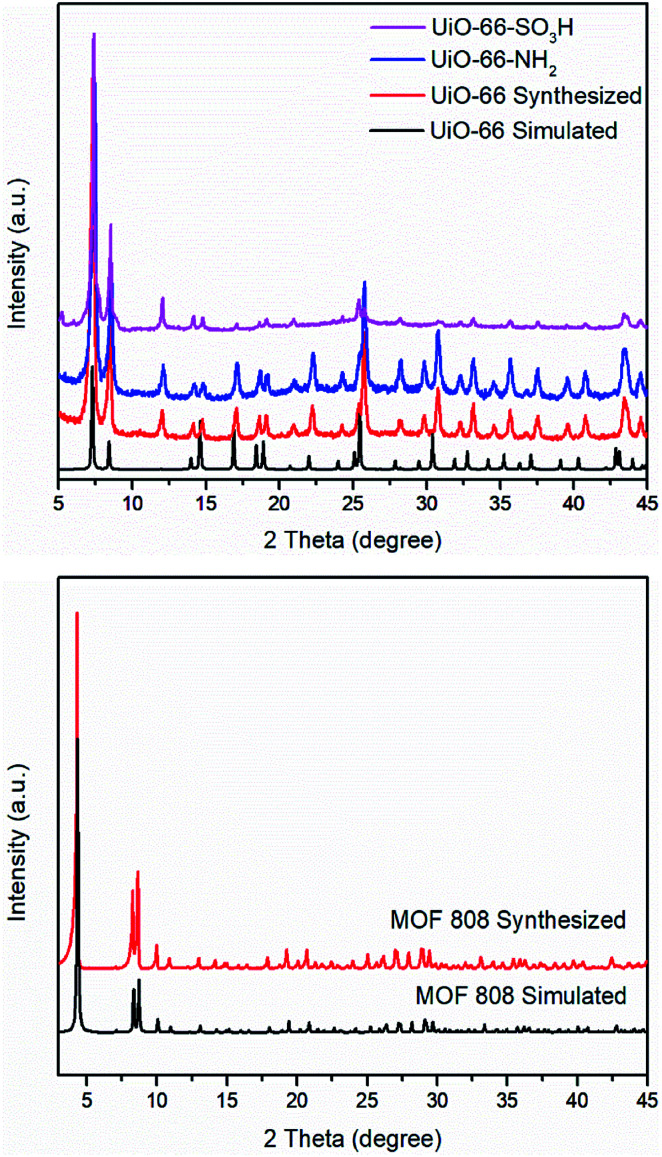
PXRD patterns of UiO-66–X (X = H, NH_2_, and SO_3_H) including the simulated pattern for UiO-66 (top) and MOF 808 including the simulated pattern of MOF 808 (bottom).

A FT-IR study of UiO-66 and its analogues reveals that FT-IR spectra of activated forms of UiO-66–X are similar (Fig. S1†). Two strong absorption bands in the region of 1560–1600 cm^−1^ and 1380–1415 cm^−1^ are attributed to carboxylate asymmetric and symmetric stretching.^25,27^ An intense absorption band is observed in the region of 1653–1665 cm^−1^ due to the C=O stretch of DMF within the pores of the MOF.^26,28^ Another medium absorption band in the region of 1495–1507 cm^−1^ is the results of CC ring symmetric stretches within the linkers.^28^ For UiO-66–NH_2_, in the high frequency region, two absorption bands can be found at 3350 cm^−1^ and 3451 cm^−1^ as the result of asymmetric and symmetric stretches of a primary amino group (–NH_2_).^29,30^ In the low frequency region, a weak N–H bending vibration at 1617 cm^−1^ and a strong C–N stretching absorption located at 1257 cm^−1^ also confirm the presence of the amino group.^29^ For UiO-66–SO_3_H, the O=S=O asymmetric and symmetric stretching bands appear at 1076 cm^−1^ and 1024 cm^−1^.^25^ PXRD patterns of UiO-66–X are shown in Fig. 3. All of these three solids retain their crystallinity and their corresponding patterns are almost same as compared with simulated UiO-66.

**Fig. 4:**
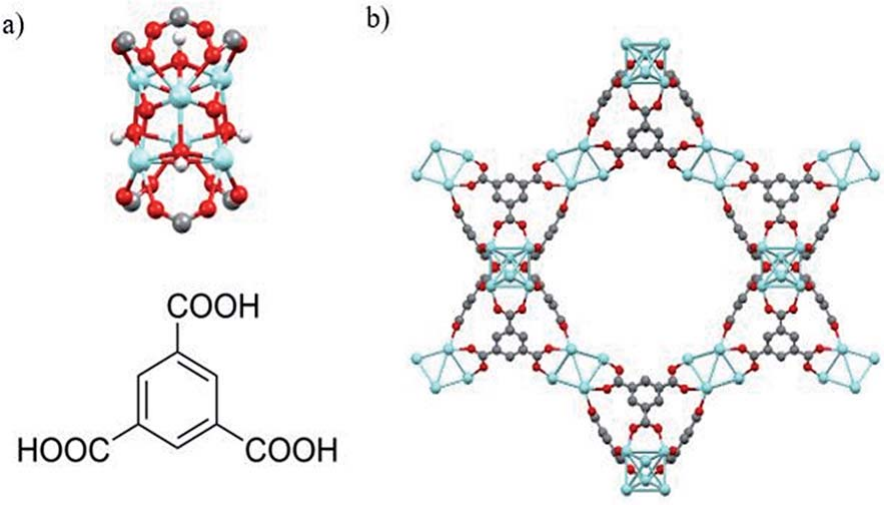
(a) Illustration of Zr_6_O_4_(OH)_4_ secondary building units (top) and trimesic acid (H_3_BTC) organic linkers (bottom). (b) Structural representation of MOF 808. Zr, blue; O, red; C, gray; H, white. μ_3_-O and H atoms are omitted for clarity.

As with UiO-66, MOF 808^23^ is built up from octahedral [Zr_6_O_4_(OH)_4_]^12+^ cationic nodes and trimesic acid (H_3_BTC) organic linkers (Fig. 4). The secondary building units are connected with six BTC linkers. Six formate ligands cap the node and provide for the anions necessary for charge balancing.^23^ Compared with UiO-66–X, MOF 808 has a larger pore size with the internal pore diameter of 18.4 Å *vs.* 6 Å for UiO-66.^23,26^ The FT-IR spectra of MOF 808 is almost the same as those reported by others (Fig. S2†).^31,32^ Two intense absorption bands at 1606 cm^−1^ and 1378 cm^−1^ are attributed to carboxylate asymmetric and symmetric stretching. PXRD study shows that the experimental pattern of MOF 808 used in the present study is almost the same as the simulated pattern (Fig. 3).

### Conversion of glucose into 5-HMF

**Fig. 5:**
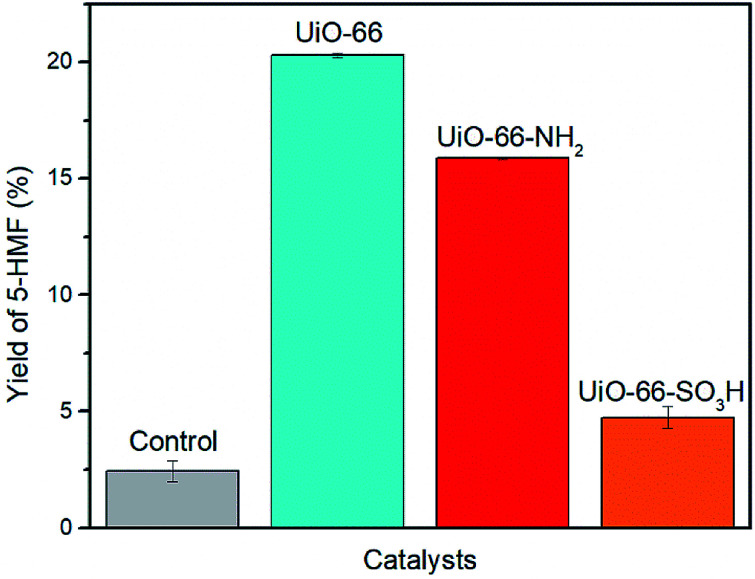
Yield of 5-HMF in the catalytic conversion of glucose to 5-HMF. Conditions: microwave, 100 mg glucose, 10 mg UiO-66–X (X = H, NH_2_, SO_3_H), 2 mL DMSO-*d*_6_, 160 °C, 20 min.

Zr-cluster MOFs are potential candidates for conversion of glucose due to their chemical stability and exceptionally high surface areas. Also, we hypothesized that the Lewis acidic Zr(iv) metal nodes could facilitate dehydration of glucose. We thought that the Brønsted acidic SO_3_H-functionalized UiO-66 could improve the yield of 5-HMF *via* bifunctional acid catalysis. In the case of UiO-66–NH_2_, we postulated that the presence of the amino group might assist in the glucose isomerization process due to its Lewis basic properties. Thus, UiO-66 and its analogues were considered as our primary catalyst targets in the dehydration of glucose. Reactions were performed in DMSO-*d*_6_ to allow monitoring of reactions using ^1^H NMR spectroscopy.^21^ Identical results were obtained in reactions performed in protio-DMSO. Fructose, a potential intermediate,^21^ was not observed in the NMR spectra of any of the reaction mixtures. In a control reaction, 5-HMF was formed in 2% yield in the absence of a MOF catalyst. We also confirmed that Zr salts or other soluble leachates were not responsible for the catalysis by heating UiO-66 in DMSO-*d*_6_ (160 °C, 20 min), filtering the solution and using this solution to perform a second control reaction. This also gave a yield of 2% – identical to that obtained using fresh DMSO-*d*_6_. We note that under the conditions presented in Fig. 5, a control (blank) reaction using fructose in the place of glucose gave a 61% yield of 5-HMF. This shows that DMSO itself is an efficient catalyst for the conversion of fructose for 5-HMF, as observed by Amarasekara *et al.* in 2008.^21^ Therefore, our catalytic studies focused solely on glucose as the substrate.

Our initial study attempted to determine the most active of the three MOFs (UiO-66, UiO-66–NH_2_ and UiO-66–SO_3_H) in the conversion of glucose to 5-HMF (Fig. 5). The initial microwave reaction was performed with a catalyst loading of 10 mg for 100 mg of glucose (*i.e.*, 10% w/w). Under identical conditions, the highest yield of 5-HMF (20%) was obtained using UiO-66 (Fig. 5).

Somewhat surprisingly, the yield of 5-HMF obtained using UiO-66–NH_2_ was 4% lower than that obtained using UiO-66 and only 5% yield was obtained using UiO-66–SO_3_H. This observation is contrary to our initial hypotheses and somewhat surprising given the similar number of accessible Lewis acidic sites available in each of these materials. We propose that the surface area of the UiO-66 materials is the critical parameter in determining reactivity and that the presence of –NH_2_ and –SO_3_H groups results in much lower surface areas (1045 m^2^ g^−1^ and 515 m^2^ g^−1^ respectively compared with 1650 m^2^ g^−1^ for UiO-66) and hence, lower yields (Table S1 and Fig. S3†). This also implies that the Lewis acidic centres within the pores (rather than only surface Lewis acid sites) are playing a key role in the isomerization step (glucose to fructose) in this reaction.

### Optimization of reaction conditions

As the initial screening showed that the highest yield of 5-HMF was obtained using UiO-66, we then tried to optimize reaction conditions to improve the yield of 5-HMF. The results for catalytic conversion of glucose to 5-HMF with different catalyst loadings at various temperatures and times are reported in Table 1 and Fig. S4.†

**Table 1:** Optimization of temperature, time and amount of UiO-66 for the conversion of glucose to 5-HMF^a^^,^^b^

Entry	UiO-66 loading (mg)	Temperature (°C)	Time (min)	Yield (%)
1	10	160	20	20
2	10	160	30	21
3	10	160	40	16
4	10	160	50	15
5	20	150	30	9
6	20	160	30	28
7	20	160	30	37^c^
8	20	170	30	26
9	20	180	30	26
10	20	190	30	16
11	30	160	30	25

a Unless stated otherwise, the dehydration reaction of glucose to 5-HMF was conducted in the presence of 100 mg glucose and 2 mL DMSO-*d*_6_.

b After reaction, 15 μL 1-naphthaldehyde was added into the reaction mixture for quantitative ^1^H NMR analysis. No glucose was observed in the spectra of any reaction mixtures (monitored by C_(2)_ OH̲ *δ* 6.2), Fig. S5 and S6.

c Reaction was conducted in 2 mL solvent mixture of DMSO-*d*_6_/water (2.5% v/v water).

#### (a) Effect of reaction time on yield of 5-HMF

**Fig. 6:**
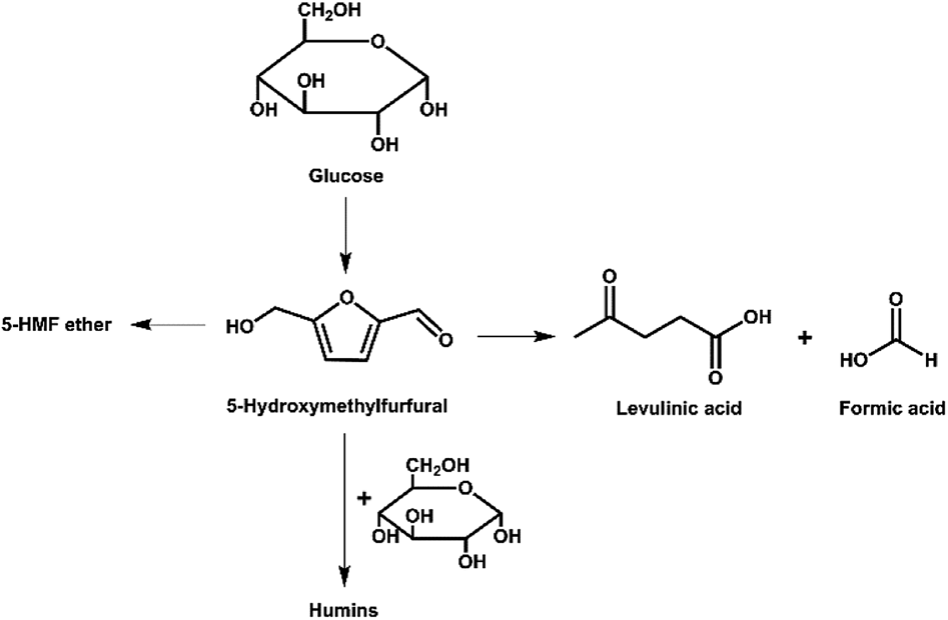
Pathways for decomposition of 5-HMF during glucose conversion.

Reaction times of 20 to 50 min were tested. The results in Table 1 and Fig. S4† demonstrate that the yield of 5-HMF increases initially and then decreases with increasing reaction time. We suspect that this is due to the decomposition of 5-HMF after 30 min; this trend has been observed by others.^33,34^ Whilst keeping UiO-66 loading and temperature constant, the highest yield (21%) of 5-HMF was obtained at 30 min (Table 1, Entry 2). As illustrated in Fig. 6, there are three typical pathways for the decomposition of 5-HMF (the rehydration of 5-HMF to levulinic acid and formic acid, as well as self- or cross-polymerization of 5-HMF).^33,35–37^ In our study, the rehydration of 5-HMF was inhibited by the presence of DMSO since less than 4% formic acid was evident by ^1^H NMR in the reaction mixtures. A similar observation was reported by Qi *et al.* in 2009.^33^ Levulinic acid was not seen in the ^1^H NMR of reaction mixtures and therefore must have reacted to form an insoluble by-product. We propose that the reduction in yield after an optimum time has passed is most likely due to polymerization of 5-HMF to form humin. A more detailed discussion of humin formation is presented below.

#### (b) Effect of catalyst loading on yield of 5-HMF

To assess the effect of different catalyst loadings on yields of 5-HMF, the reaction was conducted in the presence of 100 mg glucose at 160 °C for 30 min using 10, 20 or 30 mg of UiO-66. The yield of 5-HMF in a control blank reaction was 3% but increased to 21% when 10 mg UiO-66 was used and 28% when 20 mg was used (Table 1, Entry 6). However, the yield of 5-HMF dropped slightly to 25% when 30 mg UiO-66 was used. Dehydration of glucose is accelerated in an acidic environment.^5*b*^ Thus, more UiO-66 added to the reaction system, and consequently a greater number of Lewis acidic Zr(iv) sites, should speed up the conversion of glucose to 5-HMF and increase the yield of 5-HMF. Unfortunately, in this study the yield of 5-HMF decreases at higher catalyst loadings possibly due to side reactions (including humin formation) between glucose and 5-HMF *i.e.* the MOF-catalysed glucose isomerization to the intermediate fructose may have been sped up but so have Lewis-acid catalyzed side reactions.

#### (c) Effect of temperature on yield of 5-HMF

High temperatures are essential for the dehydration of glucose.^5*b*,33,34^ Thus, temperature was varied from 150 °C to 190 °C. As shown in Table 1 and Fig. S4,† the yield of 5-HMF increases significantly initially from 150 °C to 160 °C and reaches 28% yield of 5-HMF at 160 °C. This is a similar temperature to that reported to be optimum for the DMSO-catalyzed conversion of fructose to 5-HMF.^21^ In the current study, the yield of 5-HMF decreases at reaction temperatures above 160 °C and drops to 16% at 190 °C due to decomposition of 5-HMF, as has been reported by others.^33,34^ A small contribution to the decreased yield was also observed, *via*^1^H NMR, from the rehydration of 5-HMF at higher temperatures. Around 8% formic acid was obtained above 160 °C but no levulinic acid was seen *via*^1^H NMR. Therefore, the main cause of such low yields is the polymerization of 5-HMF to form insoluble humin by-products.

In a previous study, an optimum yield of 5-HMF could be obtained in the solvent mixture of THF/water (v/v 39 : 1) in the catalytic conversion of glucose/cellulose.^18,38^ Thus, for our reaction, the solvent mixture of DMSO-*d*_6_/water (v/v 39 : 1) was also investigated. The maximum yield (37% of 5-HMF) was obtained (Table 1, Entry 7), which was nearly 10% higher than the yield (28%) using pure DMSO-*d*_6_, under the same reaction conditions. Our results are consistent with those obtained by others, which provides further evidence that just a small amount of water can facilitate the conversion of glucose to 5-HMF, due to increased solubility of the substrate.^18,38^

It is important to compare our results with those already performed using different MOFs. Table 2 summarizes the yield of 5-HMF in the catalytic conversion of glucose to 5-HMF with three different typical MOFs. Unlike UiO-66–SO_3_H catalyst studied herein, the SO_3_H-functionalized MIL catalyst gave a good yield (29%) of 5-HMF but only 2% 5-HMF was formed using bare, unsulfontated MIL-101.^18^ The possible explanation is that MIL-SO_3_H has more Brønsted acidic sites and maintains a fairly high surface area (1333 m^2^ g^−1^) as compared with bare MIL-101.^18^ Katz, Farha and co-workers found that the phosphate-modified NU-1000 gave much higher yield of 5-HMF, comparing with the yield using bare NU-1000.^19^ Moreover, the yield of 5-HMF increased in the solvent mixture of 2-PrOH/water (v/v 9 : 1) with PO_4_/NU(half).^19,38^ They also demonstrated that a lower concentration of glucose could reduce humin formation, which enhanced the yield of 5-HMF (Table 2, Entry 8).^19^

**Table 2:** Comparison of glucose conversion to 5-HMF using UiO-66 and literature reports using three different MOFs

Entry (Ref.)	Glucose loading (mmol)	Reaction conditions				5-HMF yield (%)
		Solvent	Catalyst	Temp. (°C)	Time	
1 (this work)	0.56	DMSO-*d*_6_	UiO-66	160	30 min	28
2 (this work)	0.56	39 : 1 (v/v) DMSO-*d*_6_/water	UiO-66	160	30 min	37
3 (ref. 18)	1.24	39 : 1 (v/v) THF/water	MIL-101	130	24 h	2.0
4 (ref. 18)	1.24	39 : 1 (v/v) THF/water	MIL-SO_3_H	130	24 h	29
5 (ref. 19)	0.10	Water	NU-1000	140	5 h	2.3
6 (ref. 19 and 38)	0.10	Water	PO_4_/NU(half)	140	5 h	15
7 (ref. 19 and 38)	0.10	9 : 1 (v/v) 2-PrOH/water	PO_4_/NU(half)	140	5 h	20
8 (ref. 19 and 38)	0.10 × 10^−2^	9 : 1 (v/v) 2-PrOH/water	PO_4_/NU(half)	140	5 h	64

### Recycling test

**Fig. 7:**
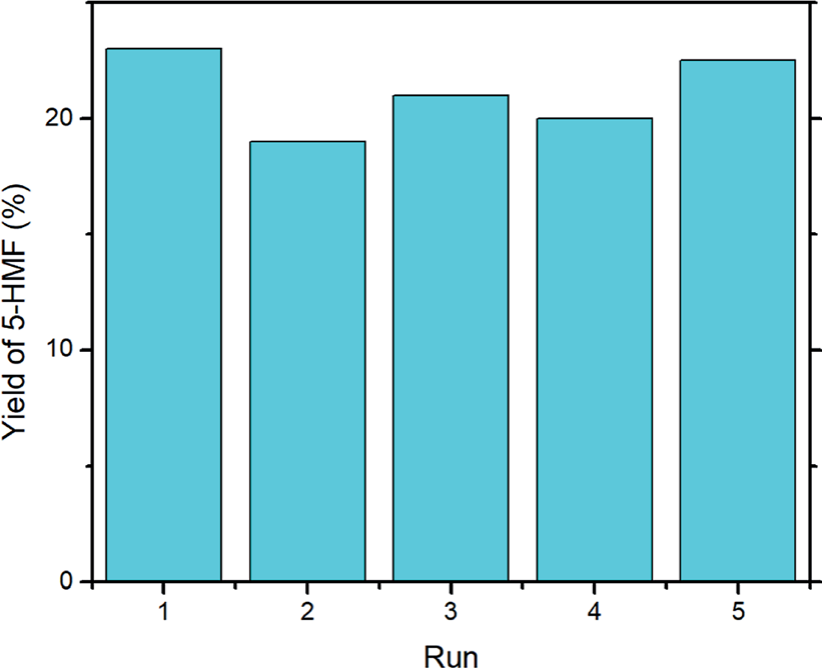
Recycling experiment with UiO-66. Reaction conditions: microwave, 1000 mg glucose, 200 mg UiO-66, 15 mL DMSO-*d*_6_, 160 °C, 30 min.

In order to evaluate the stability of UiO-66, catalyst recycling experiments were performed, and the results are reported in Fig. 7. Overall, the yield of 5-HMF decreased very slightly on first re-use (run 2, 19%) but gave a similar yield to run 1 upon its fourth re-use (run 1, 23%; run 5, 22.5%). The mean yield across runs 2–5 was 20.6%, standard deviation ± 1.5%.

**Fig. 8:**
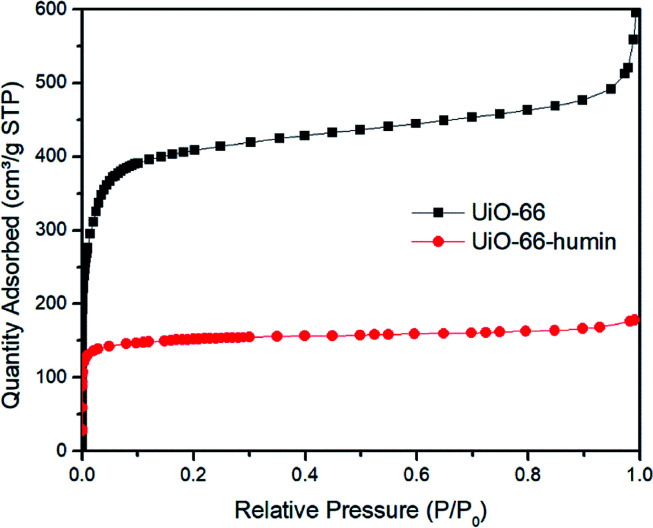
Nitrogen adsorption isotherms at 77 K of UiO-66 and UiO-66–humin.

During the course of reactions, we observed that the color of the solid catalyst changed from white to dark brown (Fig. S7†). The existence of humin on the surface or inside the pores of UiO-66 would cause a decrease in the surface area, which would affect the yield of 5-HMF. Thus, we examined the surface area of UiO-66 before and after reaction – the latter material we refer to as UiO-66–humin from herein. From N_2_ adsorption isotherms, the BET surface area for UiO-66 and UiO-66–humin was seen to decrease significantly from 1650 m^2^ g^−1^ to 598 m^2^ g^−1^ respectively (Fig. 8). Also, TGA data confirmed the presence of a significant amount of organic matter within UiO-66–humin. Between 100 and 450 °C a weight loss of 36 wt% is observed for UiO-66–humin whereas no weight loss occurs between these temperatures for UiO-66. This organic matter would account for 10 wt% of the original glucose used.

### Characterization of humin on MOF surface

Humin is an unavoidable byproduct in the catalytic conversion of biomass,^39^ and its formation can be catalyzed by Lewis acidic centres.^6*a*,*d*^ The structure of humin has not been well-studied until recently. Sumerskii proposed that humin is composed of 60% furan rings together with 20% ether or acetal aliphatic linkers.^22*b*^ Furthermore, the mechanism of humin formation is not fully understood. In the acidic dehydration of glucose using MOFs, the formation of humin has been observed after reaction by our group and others.^18,19,40^ However, as far as we are aware although papers have reported the formation of humin on MOF surfaces indirectly, *e.g.*, through surface area measurements, the characterization of humin on the surface of a MOF has not been reported previously. Herein, we tried to investigate the existence of humin on UiO-66 using different characterization techniques.

Due to the formation of humin, the color of UiO-66 changed to dark brown after glucose reactions (Fig. S5†). We assume that the existence of the humin on the surface significantly affects the yield of 5-HMF obtained. Then, we examined the surface area of UiO-66 and UiO-66–humin. Its adsorption ability decreased significantly after reaction (Fig. 8). The BET surface area of UiO-66–humin was 598 m^2^ g^−1^, which was considerably lower than that of UiO-66 before the reaction (1653 m^2^ g^−1^). These differences in the N_2_ adsorption isotherms indicate the formation of humin would inevitably affect the efficiency of UiO-66 in catalysis, assuming that the reaction proceeds *via* a heterogeneous mechanism wherein surface area will be a critical parameter for catalytic activity. We note in studies by others that similar decreases were observed.^18^ Herbst and Janiak reported that the surface area of MIL-SO_3_H was reduced from 1333 m^2^ g^−1^ to 443 m^2^ g^−1^ after reaction in the THF/water mixture of 39 : 1.^18^

**Fig. 9:**
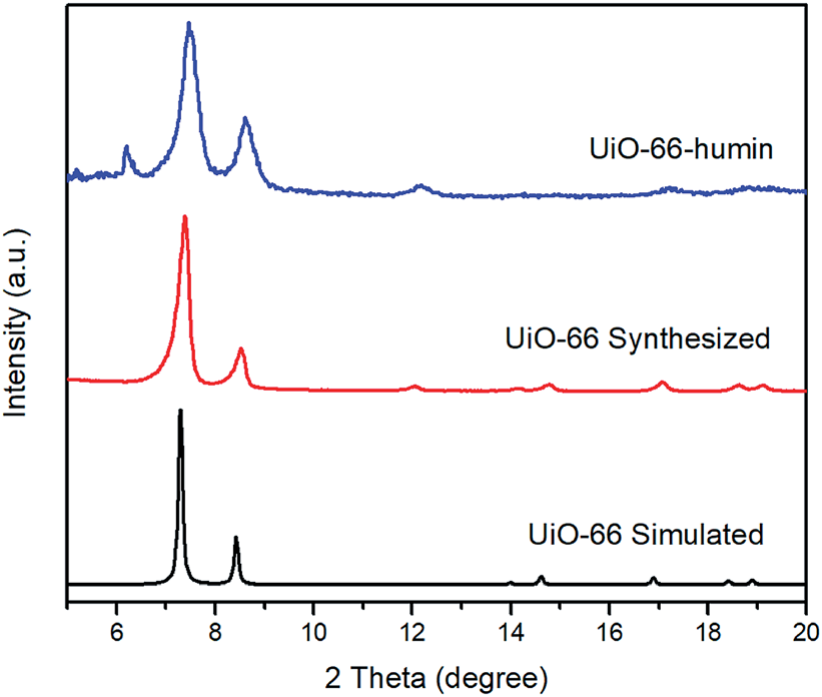
PXRD patterns of UiO-66 simulated (black), UiO-66 synthesized (red) and UiO-66–humin (blue).

PXRD patterns of simulated UiO-66, synthesized UiO-66 and UiO-66–humin show no significant changes in peak positions (Fig. 9). However, the crystallinity of UiO-66–humin is diminished with respect to UiO-66 since the two main peaks at 2*θ* 7.4° and 8.5° have become broader, and likely less intense (as evidence by the signal-to-noise ratio) after reaction. We hypothesize that the occurrence of an additional small peak at a 2*θ* of 6.2° in UiO-66–humin represents ‘forbidden’ reflections for the topological space group due to diffuse scattering by the humin.^41^

Using FT-IR spectroscopy, we find that the bands in the spectrum for UiO-66–humin are much broader than those in the spectrum of UiO-66 (Fig. S8†). In the spectrum of UiO-66–humin, a broad peak around 3352 cm^−1^ can be attributed to C–O stretch from alcohols.^22*b*,42^ A weak absorption band at 2919 cm^−1^ is from aliphatic C–H stretches.^22*b*,42^ Some differences between the spectra of these materials might be attributed to the existence of furan rings, such as the broader C=C stretching absorption at 1583 cm^−1^ and the C–O stretching absorption at 1017 cm^−1^,^42^ with the latter band being significantly more intense than a weak absorption in the same region for UiO-66. Below 1000 cm^−1^ in the fingerprint region, peaks at 952 cm^−1^ and 746 cm^−1^ might also indicate the presence of substituted furan rings.^22*c*^

**Fig. 10:**
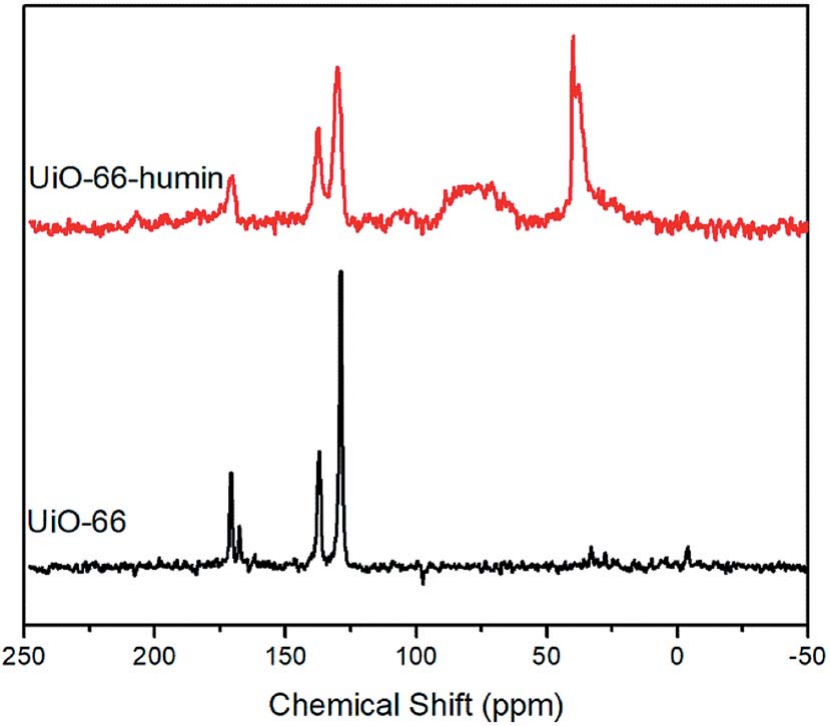
^13^C solid state NMR spectrums of UiO-66 (bottom) and UiO-66–humin (top).

To further investigation the formation of humin on UiO-66, ^13^C solid-state NMR spectroscopy was applied to examine the UiO-66 samples before and after reaction (Fig. 10). The ^13^C solid-state NMR spectrum of UiO-66 contains three characteristic peaks at chemical shifts of 128.9, 137.1 and 170.8 ppm. Based on the study by Devautour-Vinot and Martineau *et al.*,^43^ the peak at 128.9 ppm is ascribed to the –CH group of the aromatic rings. The peak at 137.1 ppm is from the quaternary aromatic carbon atoms. The peak at 170.8 ppm is assigned to carbon atoms from the carboxylate groups.^43^ The appearance of a low-intensity peak at 167.6 ppm is due to the C=O group of DMF solvent molecules present in the pores of the MOF. Comparing the ^13^C Solid State NMR spectrum of UiO-66–humin with UiO-66, significant peak broadening is observed in the spectrum, which indicates the formation of a material that is less crystalline, corroborating the PXRD data (Fig. 9).

Moreover, an intense peak located around 39 ppm represents the presence of tertiary and/or quaternary aliphatic carbons.^22*d*^ A broad signal between 60 and 90 ppm is the result of many different C–O groups from alcohol or ether functionalities within the humin structure.^22*c*,*d*^ These two mid-to-high field peaks, that are not found in the spectrum of UiO-66, strongly confirm the formation of humin on UiO-66 during glucose dehydration reactions. It should be noted that the peaks at ∼130, 138 and 170 ppm are broad and although these are in a similar place to the carbon atoms of the 1,4-benzene-dicarboxylate (BDC) linker in the spectrum of UiO-66, they might be coincident with substituted carbon atoms of furan rings and carbon atoms from carboxyl or ester groups in the humin.^22*d*^

We also set up three comparative reactions for studying the effect of humin formation on the yield of 5-HMF and to assess inhibitory affect that the humin has by blocking access to pores within the MOF. Initially, we ran a microwave reaction with 20 mg UiO-66 at 160 °C for 30 min – this reaction affords a yield of 28%. After that, we explored two different routes. Either 20 mg of fresh UiO-66 or 100 mg of glucose was added into the reaction system and the vial heated under the same optimal reaction conditions for 30 min. We found that the yield of 5-HMF increased from 28% to 35% when 20 mg of fresh UiO-66 was added – this suggests that some unreacted glucose and intermediate dehydration products are still present in the initial reaction mixture but unable to react to form 5-HMF once UiO-66–humin has formed. In contrast, the overall yield of 5-HMF decreased from 28% to 23% when an additional 100 mg glucose was added after the initial 30 min reaction. We think the additional 100 mg glucose could possibly cross-polymerize with the 5-HMF to produce insoluble humins and the used UiO-66 with a diminishing surface area may not catalyze the isomerization of the additional glucose effectively. In a third study, a reaction was performed using 40 mg UiO-66 for 2 × 30 min at 160 °C and a 5-HMF yield of only 26% obtained. This indicates that humin formation reduces the amount of 5-HMF that can form and that this is likely due to the humin blocking the pores of the MOF. This provides further evidence for the Lewis catalyzed reaction occurring within the pores of the MOF, which was previously suggested by the lower yields obtained using UiO-66–SO_3_H that had a lower surface area.

### Dehydration of glucose to 5-HMF using MOF 808

Another zirconium-containing MOF, MOF 808, has emerged as a potential candidate for gas adsorption^44^ and catalysis^45^ due to its unique features. Compared with 12-connected or 8-connected MOFs,^23^ MOF 808 possesses lower connectivity (6-connected) and therefore it allows greater pore access to more reactant or adsorbent molecules. Therefore, owing to its larger surface area and wider pore size, we speculated that MOF 808 (6-connected) may enhance the yield of 5-HMF obtained from glucose compared with UiO-66.

A sample of MOF 808 was examined by PXRD (Fig. 3) and N_2_ adsorption (Fig. S3†) before use. The PXRD pattern of synthesized MOF 808 shows good crystallinity as compared to the pattern of simulated MOF 808. The BET surface area of MOF 808 was 1970 m^2^ g^−1^ (Table S1†). The catalytic reaction was performed at 160 °C for 30 min. MOF 808 affords the highest yield of 5-HMF (31%) of the four MOF catalysts studied herein under our standardized conditions. This yield is 10% higher than that produced by UiO-66 (21%) under identical conditions. We propose that the higher yield produced by MOF 808 can be attributed to its large pore size and high surface area. However, it may also be due to lower coordination numbers of the zirconium centres, which means they are can interact more readily with substrates in the catalytic cycle. The yield of 5-HMF was 28% in the presence of the solvent mixture of DMSO-*d*_6_/water (v/v 39 : 1), which is slightly lower than the yield obtained using pure DMSO-*d*_6_. We hypothesize that the aggregation of water molecules in the pores of MOF 808 might block the channels of MOF 808 and thereby decrease the yield of 5-HMF.^23^ This is more likely to occur in MOF 808 compared with UiO-66, as the Zr centres in MOF 808 are coordinatively unsaturated compared with those in UiO-66 and this means the water molecules are more likely to coordinate at the Zr centres and block the pores or prevent coordination of the glucose to the Lewis acid sites. Further studies are therefore needed to fully understand the interplay of surface area and coordination site availability in such catalyst systems. We considered studying the related zirconium-containing MOF, UiO-67, which contains biphenyldicarboxylate linker units in place of terephthalate units.^9*d*^ However, due to the known poor hydrolytic stability of the metal–carboxylate bonds in UiO-67,^46^ we did not pursue this.

Unfortunately, MOF 808 turned dark brown after reaction, which indicated humin formation. Herein, MOF 808 after reaction is named as MOF 808-humin. Using FT-IR spectroscopy, we compare spectrums of MOF 808 and MOF 808-humin (Fig. S9†). In the spectrum of MOF 808-humin, a broad peak around 3340 cm^−1^ is due to C–O stretch from alcohols.^22*b*,42^ Other differences between these two also indicate the formation of furan rings, for instance, the C=C stretching absorption at 1577 cm^−1^ and the C–O stretching absorption at 1023 cm^−1^.^42^ Moreover, below 1000 cm^−1^ in the fingerprint region, peaks at 990 cm^−1^ and 758 cm^−1^ are attributed to the substituted furan rings.^22*c*^ We find that some similarities between UiO-66–humin and MOF 808-humin demonstrate that the structure of humin contains furan rings with alcohol functional groups (Fig S10†).

Unfortunately, in attempts to re-use MOF 808, recycling was less successful than with UiO-66. Upon re-use of MOF 808 for dehydration of glucose, in a similar fashion to that described above using UiO-66, the yield for run 2 was only 14% (compared with 38% in run 1, and 19–22.5% in runs using recycled UiO-66). This implies that this higher surface area MOF is less stable to the hydrolytic conditions present in this transformation – hydrolytic instability has been studied previously for other zirconium-containing MOFs.^46^

We also attempted the conversion of sucrose to 5-HMF with MOF 808. The reaction conditions were the same as those used in the glucose conversion to 5-HMF with MOF 808. The yield of 5-HMF is 46%, which is 8% higher than that formed in sucrose conversion in a control reaction (38%) without added catalyst wherein DMSO would catalyze the conversion. This implies that the added MOF catalyst is playing a minor role in affording the good yield of 5-HMF from sucrose. Also, in sucrose conversion to 5-HMF, it is interesting that there is no significant difference in the yield of 5-HMF using UiO-66 (44%) and MOF 808 (46%). Based on the higher yields in these reactions compared with those of glucose, we hypothesize that intermediates particular to glucose conversion (either from glucose itself or from hydrolysis of sucrose) rather than fructose conversion are responsible for humin formation on the surfaces of the MOF catalyst and inhibit its reactivity. During the course of our research, it has been reported that UiO-66 is an efficient catalyst for isomerizing glucose to fructose in alcoholic media.^47^ Therefore, solvent choice is critical to the outcome of these reactions too and the presence of alcohol would likely suppress formation of humin on the catalyst surface.

## Conclusions

UiO-66 provides the highest yield of 5-HMF among UiO-66–X compounds (X = H, NH_2_, SO_3_H). Time, temperature and catalyst loading were varied to determine the optimal reaction conditions. Recycling tests show that the catalytic efficiency of UiO-66, although only moderate, can be maintained over 5 runs. Moreover, we were able to compare UiO-66 and UiO-66–humin using different analytical techniques, which proved the existence of humin on UiO-66. The related MOF, MOF 808, which has a higher surface area and more accessible zirconium centres, gave a significantly higher yield of 5-HMF in comparison with UiO-66–X (X = H, NH_2_, SO_3_H) under identical conditions. These data suggest that, although Lewis acidic centres are critical to the glucose isomerization step of this process, surface area is a critical parameter for efficient MOF-catalyzed dehydration of glucose to yield 5-HMF, and additional functionality does not enhance catalysis as the presence of –SO_3_H or –NH_2_ functional groups leads to lower surface area materials. Further studies would be needed to fully understand the role of solvent, the interplay of Lewis acid and Brønsted acidic sites in such catalysts and confirm the importance of surface area and pore-accessibility in MOF-catalysts for this and related reactions.

## Conflicts of interest

There are no conflicts to declare.

## Supplementary Material

RA-008-C8RA06021E-s001
