# Supplementary material for: Catalytic conversion of glucose to 5-hydroxymethylfurfural using zirconium-containing metal–organic frameworks using microwave heating
Source: RSC Adv. 2018 Sep 10;8(55):31618–27. doi: 10.1039/c8ra06021e (PMC9085719; doi:10.1039/c8ra06021e)
Supplement: RA-008-C8RA06021E-s001 [file RA-008-C8RA06021E-s001.pdf]

Electronic Supplementary Material (ESI) for RSC Advances.
This journal is © The Royal Society of Chemistry 2018

Electronic Supplementary Information

Catalytic Conversion of Glucose to 5-Hydroxymethylfurfural using Zirconium-Containing Metal-Organic Frameworks using Microwave Heating

Jue Gong, Michael J. Katz* and Francesca M. Kerton*
Department of Chemistry, Memorial University of Newfoundland, St. John's, NL A1B 3X7, Canada.

E-mail: fkerton@mun.ca, mkatz@mun.ca

Materials and Reagents

All reagents were purchased and used as received. *N,N′*-dimethylacetamide (HPLC grade) and hydrochloric acid (ACS reagent) were purchased from Caledon Laboratories Ltd. *N,N′*-dimethylformamide (ACS reagent, ≥ 99.8%) methanol (ACS reagent) and dimethyl sulfoxide (ACS reagent, ≥ 99.9%) were purchased from ACP Chemicals Inc. D-fructose (99%) was purchased from Alfa Aesar. Formic acid (98%) was purchased from Fluka. Monosodium 2-sulfoterephthalate (>98%) was purchased from TCI. Dimethyl sulfoxide-*d6* (D, 99.9%) + 0.05% v/v TMS was purchased from Cambridge Isotope Laboratories, Inc. Sucrose is table sugar from the market. The following reagents were purchased from Sigma-Aldrich: Zirconium(IV) chloride (≥ 99.5%), Zirconium(IV) oxychloride octahydrate (≥ 99.5%), terephthalic acid (98%), 2-aminoterephthalic acid (99%), trimesic acid (95%), α-D-glucose (96%), and 1-naphthaldehyde (95%).

Synthesis of MOFs

MOFs were synthesized using literature methods and characterization data were in good agreement with those previously reported.

UiO-66 and UiO-66-NH_2[1,2]: $ZrCl_4$ (125 mg, 0.54 mmol) was dissolved in a mixture of concentrated HCl (1 mL) and 5 mL of *N,N′*-dimethylformamide (DMF). The mixture was sonicated for 20 min. Then, terephthalic acid (123 mg, 0.75 mol) or 2-amino-terephthalic acid (134 mg, 0.75 mmol) and another 10 mL of *N,N′*-dimethylformamide (DMF) were added. The mixture was sonicated for a further 20 min. After dissolution, the mixture was heated at 80 °C overnight in an oven. Upon cooling to room temperature, the resulting solid was filtered and washed with *N,N′*-dimethylformamide (DMF) (2 × 30 mL) and then with methanol (2 × 30 mL). For UiO-66, the white precipitate was filtered. For UiO-66-NH_2, the resulting solid was refluxed in methanol at 60 °C in an oil bath overnight and then collected through vacuum filtration resulting in a pale yellow powder. Finally, both MOFs were dried at 80 °C in a vacuum oven overnight.

UiO-66-SO_3H[3]: A mixture of $ZrOCl_2 \cdot 8H_2O$ (100 mg, 0.31 mmol), BDC-SO_3Na (83 mg, 0.31 mmol) and formic acid (1.17 mL) was dissolved in 3 mL *N,N'*-dimethylacetamide (DMA). The mixture was sonicated until full dissolution then was heated at 150 °C for 24 h in an oven. After cooling to room temperature, the white solid was filtered and dried in air. Then, the as-synthesized product was heated at 65 °C in a vacuum oven for 24 h.

MOF 808[4]: H_3BTC (110 mg, 0.50 mmol) and $ZrOCl_2 \cdot 8H_2O$ (160 mg, 0.50 mmol) were dissolved in a solvent mixture of DMF/formic acid (20 mL/20 mL). Then, the mixture was heated at 100 °C for 7 days in an oven. After cooling to room temperature, the resulting solid was filtered with *N,N'*-dimethylformamide (DMF) (3×10 mL) and dried at 100 °C for 24 h.

Table S1. BET surface area of MOF compounds

Compound	BET surface area (m^2g^{-1})
UiO-66	1650
UiO-66-NH_2	1045
UiO-66-SO_3H	515
MOF 808	1970

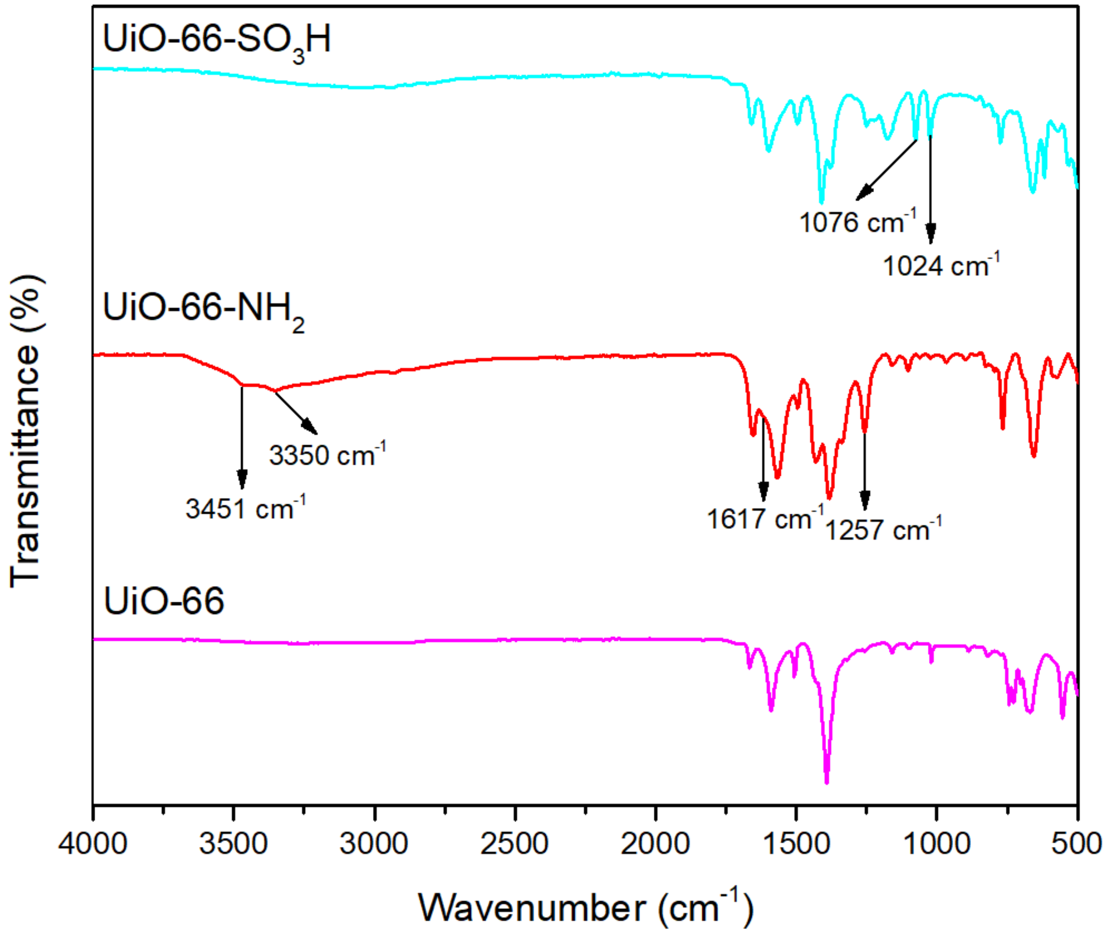

Fig. S1 FT-IR spectra of the activated UiO-66-X compounds.

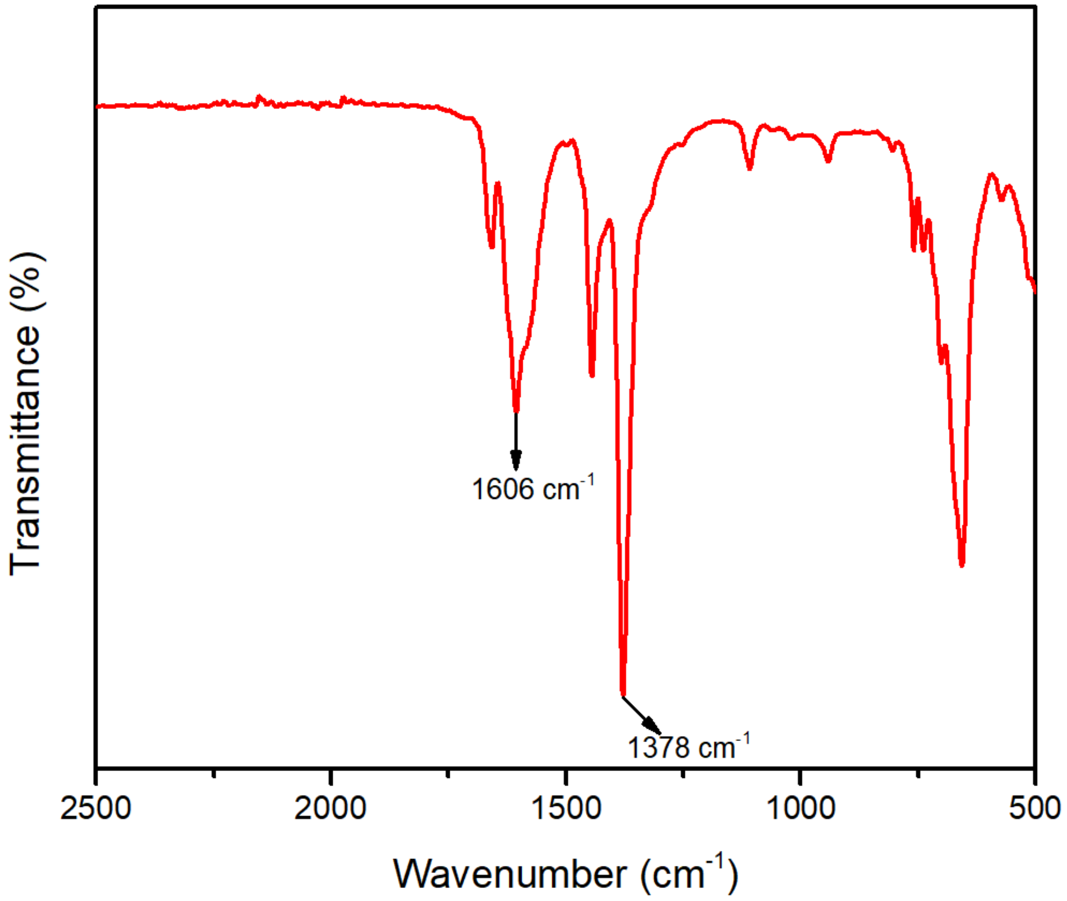

Fig. S2 FT-IR spectra of MOF 808.

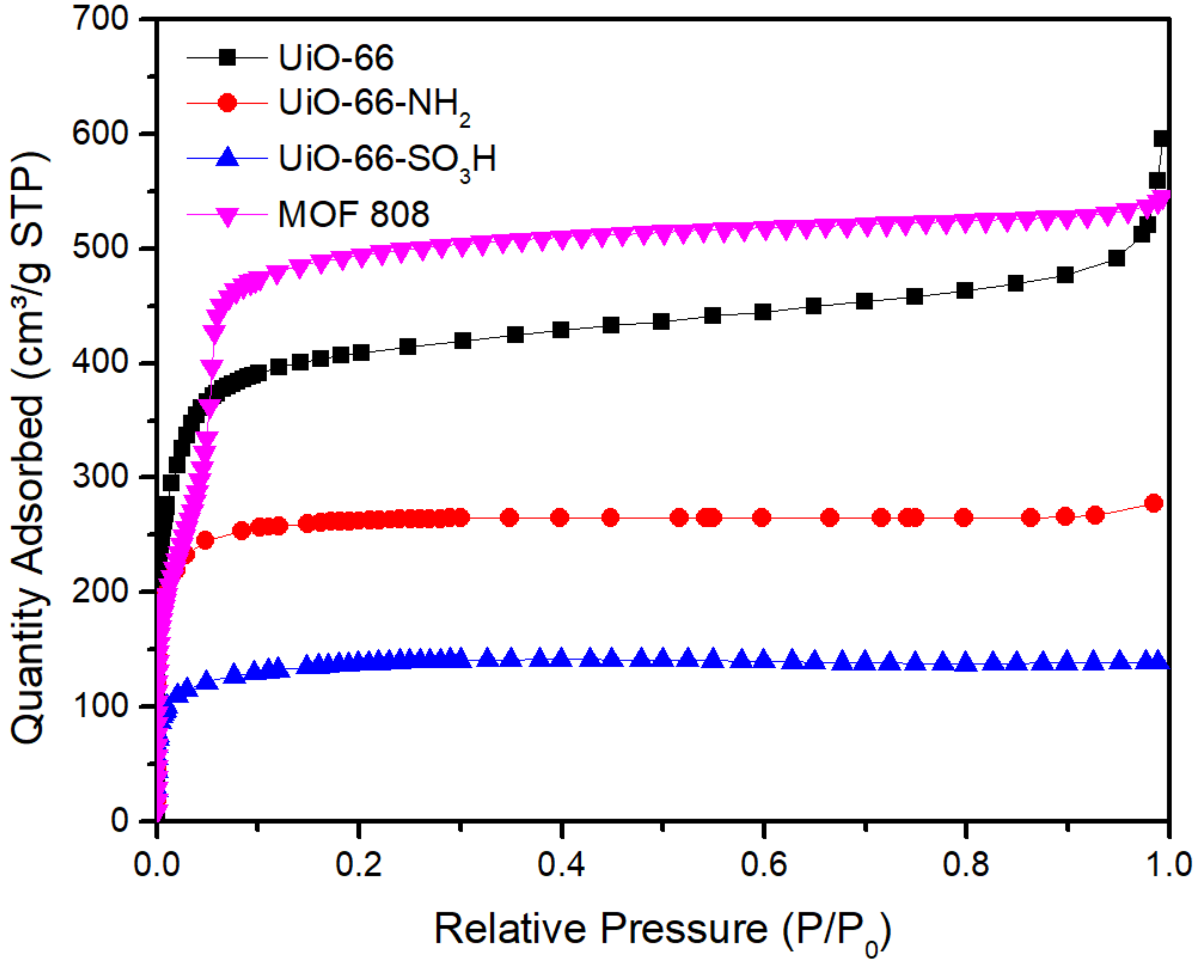

Fig. S3 Nitrogen adsorption isotherms at 77 K of UiO-66-X and MOF 808.

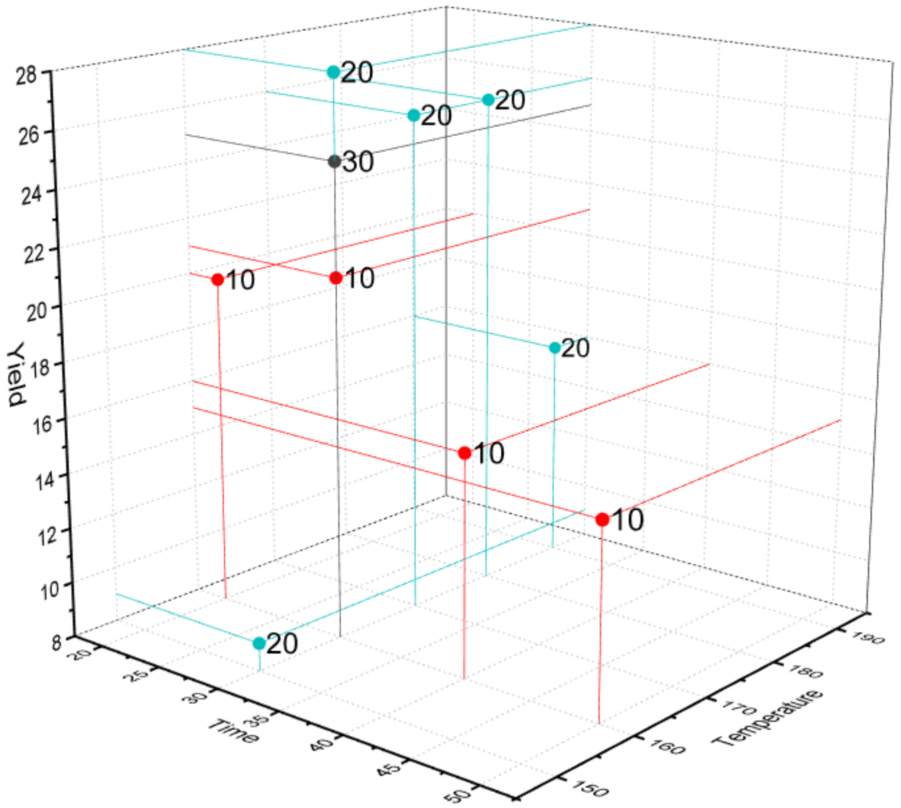

Fig. S4 3D scatter plot of yield of 5-HMF as a function of both time and temperature. Different color dots represent different catalyst loadings of UiO-66. Red, 10 mg; Blue green, 20 mg; Dark gray, 30 mg.

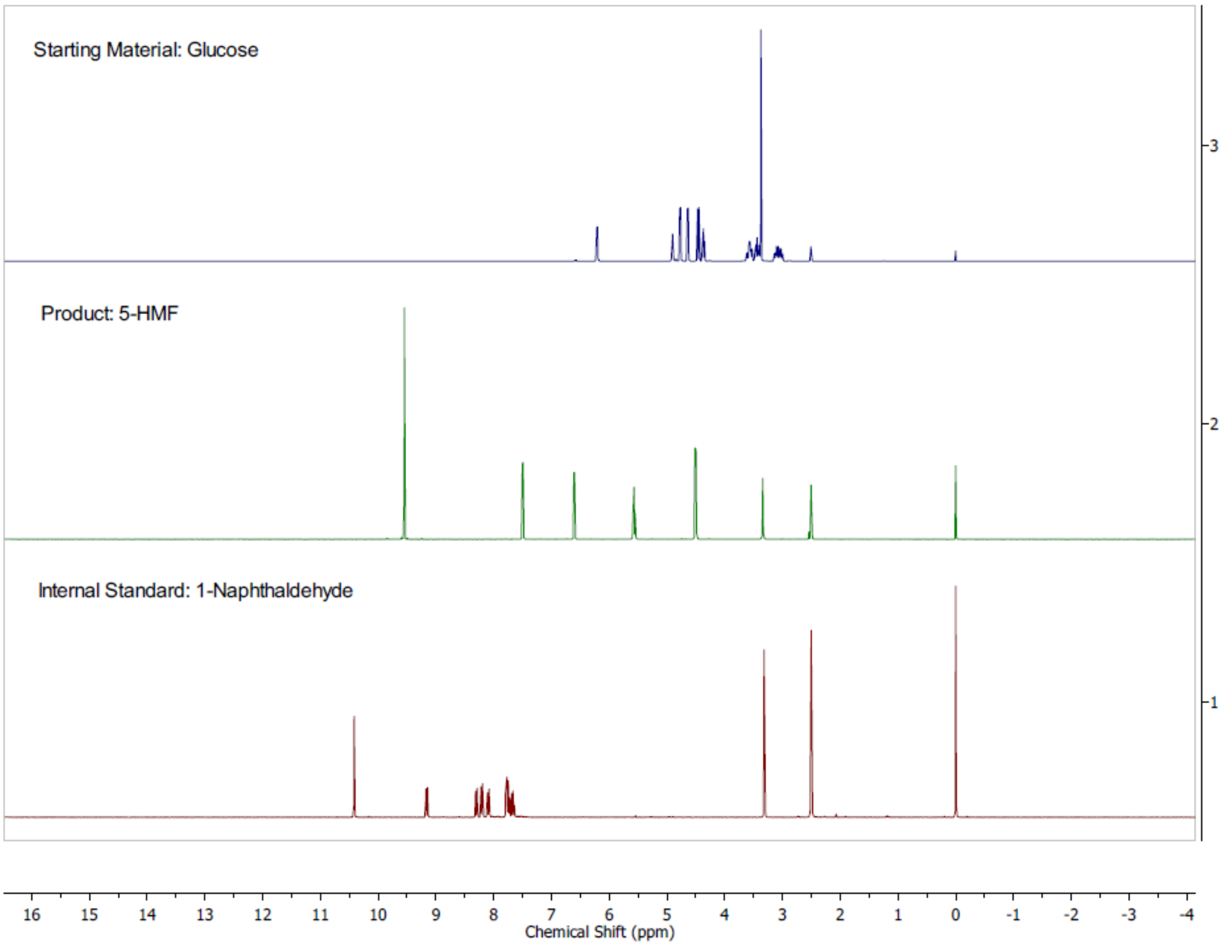

Fig. S5 Stacked ^{1}H NMR spectra of starting material (top), product (middle), and internal standard (bottom) in DMSO-*d6*.

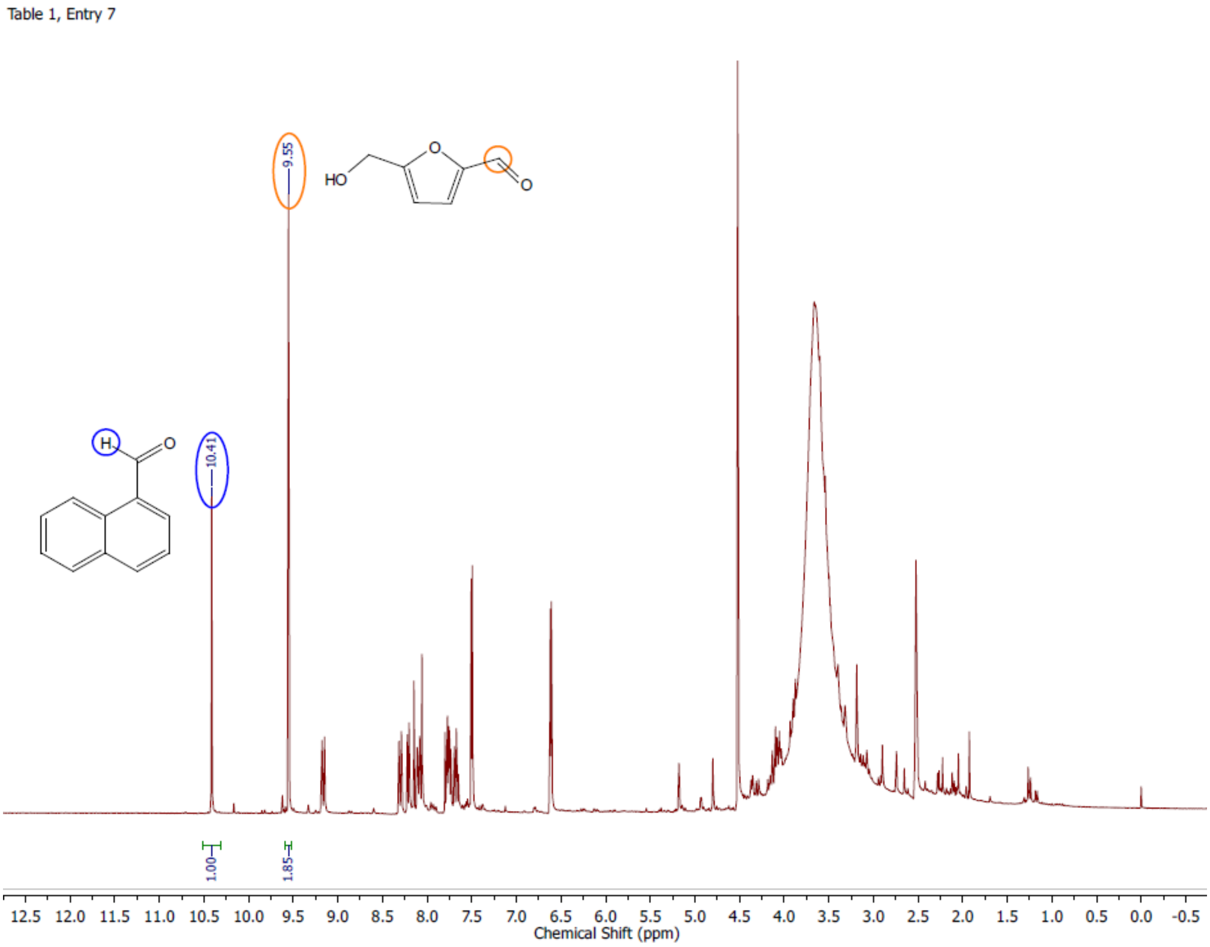

Fig. S6 Example ^{1}H NMR spectrum of reaction mixture in DMSO-*d6* (conditions as detailed in Table 1, Entry 7).

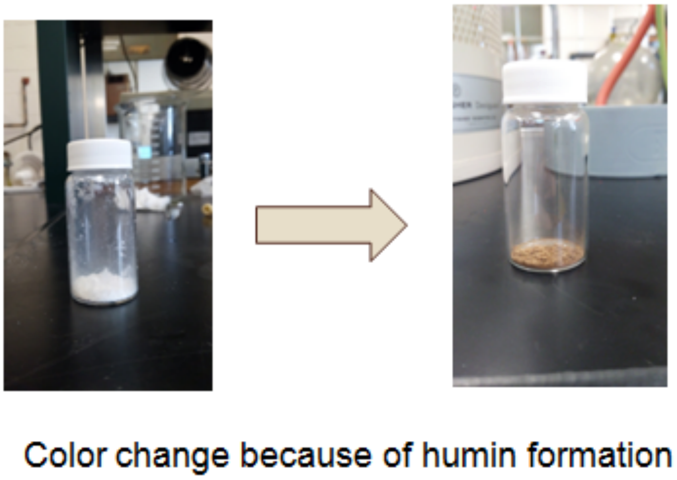

Fig. S7 Color of UiO-66 before and after conversion of glucose to 5-HMF.

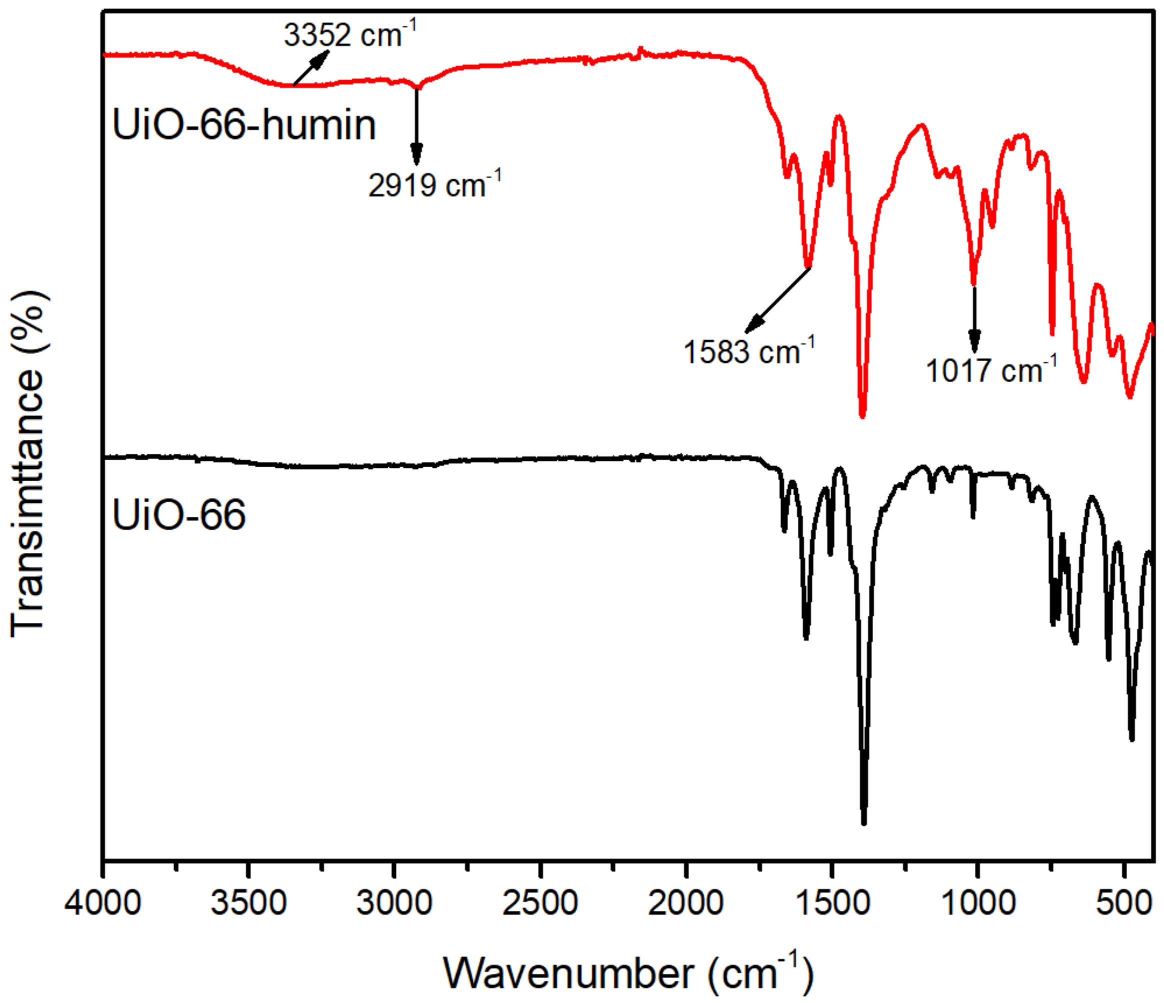

Fig. S8 FT-IR spectra of UiO-66 and UiO-66-humin.

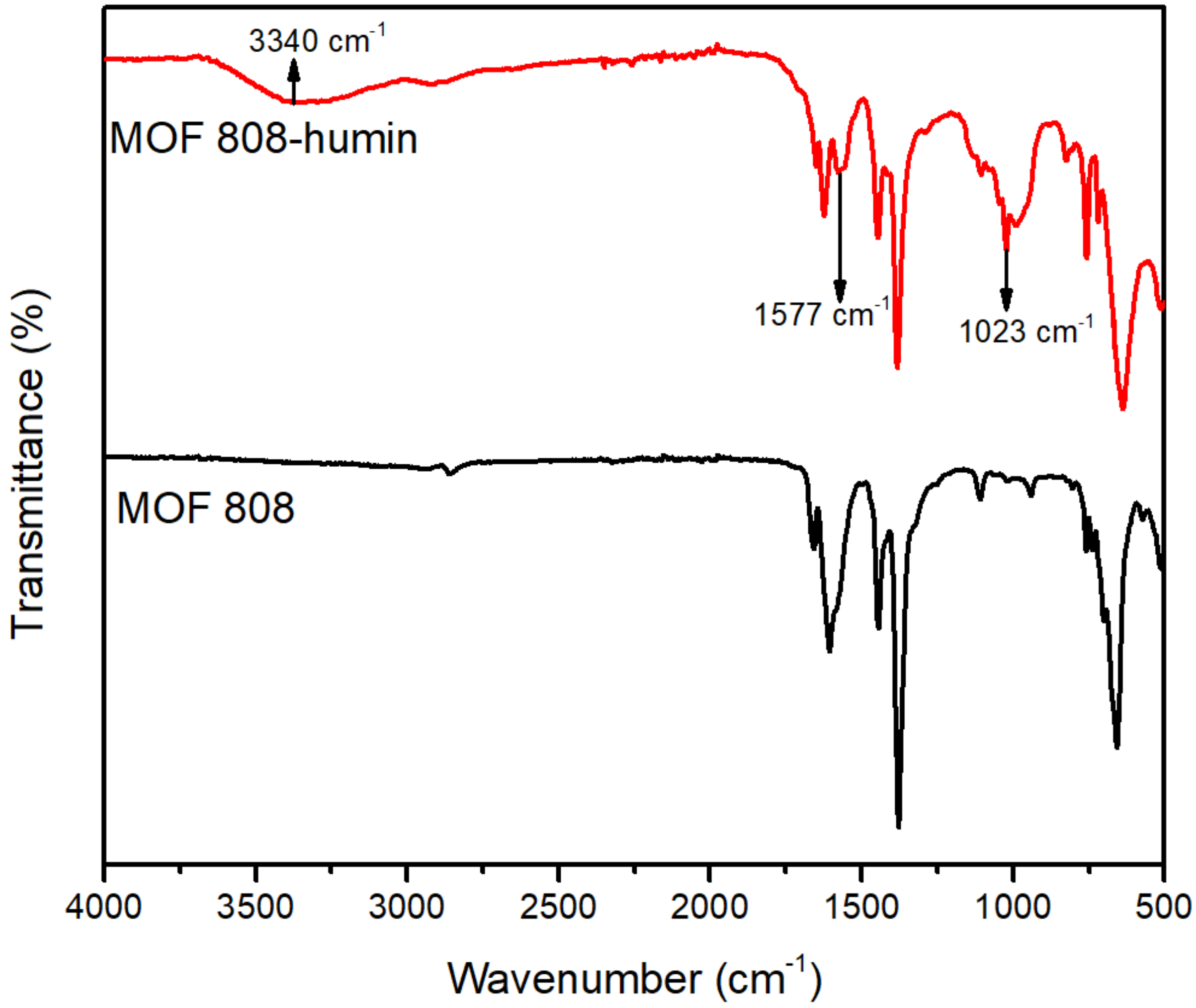

Fig. S9 FT-IR spectra of MOF 808 and MOF 808-humin.

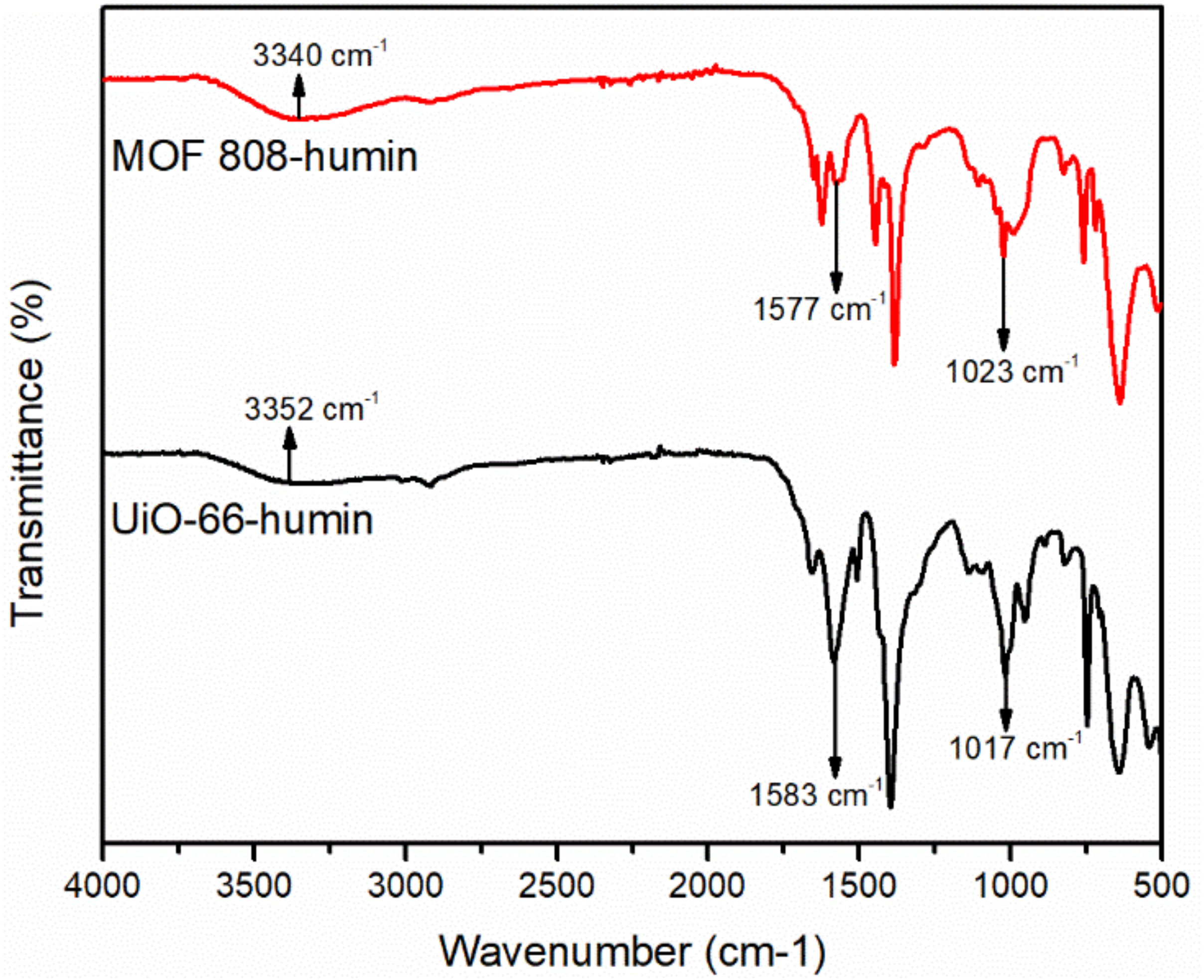

Fig. S10 FT-IR spectra of UiO-66-humin and MOF 808-humin.

References

1 M. J. Katz, Z. J. Brown, Y. J. Colon, P. W. Siu, K. A. Scheidt, R. Q. Snurr, J. T. Hupp and O. K. Farha, *Chem. Commun.*, 2013, **49**, 9449-9451.

2 K. M. Zwolinski, P. Nowak and M. J. Chmielewski, *Chemical Communications*, 2015, **51**, 10030-10033.

3 S. Biswas, J. Zhang, Z. Li, Y.-Y. Liu, M. Grzywa, L. Sun, D. Volkmer and P. Van Der Voort, *Dalton Trans.*, 2013, **42**, 4730-4737.

4 H. Furukawa, F. Gándara, Y.-B. Zhang, J. Jiang, W. L. Queen, M. R. Hudson and O. M. Yaghi, *J. Am. Chem. Soc.*, 2014, **136**, 4369-4381.
